# Financial Changes and Health During COVID-19 in the National Health and Aging Trends Study

**DOI:** 10.1093/geroni/igab046.2160

**Published:** 2021-12-17

**Authors:** Melissa Hladek, Thomas Cudjoe, Brittany Drazich, Qiwei Li, Sarah Szanton, Laura Samuel

**Affiliations:** 1 Johns Hopkins School of Nursing, Baltimore, Maryland, United States; 2 Johns Hopkins University School of Medicine, Baltimore, Maryland, United States; 3 Johns Hopkins University School of Nursing, Baltimore, Maryland, United States; 4 Johns Hopkins University, Baltimore, Maryland, United States

## Abstract

This study tested associations between income decline and financial difficulty with mental health (lack of feeling anxious/depressed, recurring thoughts/nightmares, avoiding activities/thoughts, feeling jumpy/on guard) and sleep quality during COVID-19 among a national sample of 3,188 older adults. Approximately 8% of US older adults reported income decline and 6% reported financial difficulty. Although income decline and financial difficulty rates were both statistically significantly higher among those financially strained before COVID-19 (19% and 34%, respectively), income decline was more common among those with incomes ≥200% of the poverty threshold (9%) whereas financial difficulty was more common among those with incomes <200% poverty (10%). Adjusting for sociodemographic, health and depressive symptoms before COVID-19, financial difficulty was associated with worse mental health (b= -2.39, p<0.001) and sleep quality (b=-0.820, p<0.001), but income loss was not (b= -0.685, p=0.092 and b= -0.405, p=0.082, respectively). Timely interventions are needed for older adults reporting COVID-19 financial difficulty.

